# Impact of Mobile Phone Screen Exposure on Adolescents’ Cognitive Health

**DOI:** 10.3390/ijerph191912070

**Published:** 2022-09-23

**Authors:** Monica Cristina Poujol, Ariadna Pinar-Martí, Cecilia Persavento, Anna Delgado, Monica Lopez-Vicente, Jordi Julvez

**Affiliations:** 1 Facultat de Medicina i Ciències de la Salut, Universitat de Barcelona (UB), 08036 Barcelona, Spain; 2 Clinical and Epidemiological Neuroscience (NeuroÈpia), Institut d’Investigació Sanitària Pere Virgili (IISPV), 43204 Reus, Spain; 3 ISGlobal-Instituto de Salud Global de Barcelona Campus MAR, Parc de Recerca Biomèdica de Barcelona (PRBB), 08003 Barcelona, Spain

**Keywords:** mobile phone screen exposure, attention, fluid intelligence, working memory, adolescence

## Abstract

There is existing evidence on how excessive screen exposure can be detrimental to cognitive health, and in recent years there has been an increase in the usage of mobile phones by adolescents. We aimed to examine the association between mobile phone screen exposure and cognitive function among a young healthy population. We carried out a cross-sectional study conducted in 632 adolescents (13.89 ± 0.52 years old). Exposure data were collected through self-reported questionnaires, and cognitive outcomes were assessed by different computer-based neuropsychological tests. Compared to students in the lowest tertile (<9 min/day) of mobile phone screen exposure (MPSE), those in the medium tertile (9–20 min/day) showed significantly higher hit reaction time standard error (HRT-SE, higher inattentiveness) = (14.9 ms, 95% CI = 0.6; 29.3), as did as those in the highest tertile (>20 min/day) = (11.1 ms, 95% CI = 2.8; 25.0). When adjusting for confounders, the association held for the medium-MPSE tertile (17.6 ms, 95% CI = 3.4; 31.7). When further adjusting for intermediate factors, an increase in inattentiveness scores was also observed in both groups, with higher HRT-SE values for participants in the medium (15.8 ms, 95% CI = 1.4; 30.3) and highest MPSE tertiles (14.97 ms, 95% CI = 0.9; 29.1). There were no significant associations with fluid intelligence or working memory scores. Overall, our study shows that healthy teenagers reporting higher screen exposure may be affected in their attention performance. However, more studies are needed to determine the causality of these associations and to better shape the screen exposure recommended guidelines for brain health during adolescence.

## 1. Introduction

Adolescents’ exposure to screens is currently undergoing intense study, and some evidence has been provided on how excessive exposure times can be detrimental, not only to their cognitive health but also their overall wellbeing [1]. A deficient cognitive performance can lead to unfavorable academic achievement, which in turn can lead to limited occupational choices in the future and prevent breaking out of poverty cycles [2]. The choice to spend excessive time in front of screens can provide incentives to adopt lifestyle practices that are detrimental to health, such as sedentarism; nutritional deficiencies or lower indicators of a healthy diet, such as the Mediterranean diet; and disrupted sleep patterns, which in turn can eventually lead to the development of chronic diseases that place a heavy burden on the individual and society [3].

In recent years, there has been an increase in the usage of mobile phones by adolescents [4,5,6,7,8]. It could even be stated that it is a central part of their lives [3]. According to a study by the School of Physiotherapy and Exercise Science at Curtin University in Australia, there have been several large surveys that have reported a higher prevalence of mobile phone usage in comparison to the usage of other screen devices (laptops, TVs, desktop computers, and tablets) [3]. A reason behind this might be due to the easy access to the internet and other functions that mobile phones offer (i.e., social media, messaging, games, and netsurfing) as well as the portability aspect that cell phones are known for, which makes them more convenient to use than other screen devices [3,4,6,8]. Children of lower-income families have been reported to have increased exposure to screen time in the form of TV viewing, while in higher socioeconomic families there is an increase in screen exposure through other digital devices such as mobile phones and tablets [3].

The current recommended time for youth devoted to screen time is no more than 2 h per day according to the American Academy of Pediatrics (AAP), the Canadian Pediatric Society (CPS), and the Australian Department of Health (ADH) [6]. Despite these guidelines, the vast majority of adolescents (70–80%) exceed the recreational screen-time recommendations [8]. It is estimated that adolescents spend approximately 4 h per day engaged in leisure-time sedentary screen-based behavior (SSBB), which is alarming, considering the health effects that a sedentary lifestyle can cause in the long run, along with mental health concerns [9]. In fact, excessive smartphone use has been linked with impaired cognitive functions and mental health problems among children, adolescents, and young adults [10,11]. Some longitudinal studies have found that more frequent mobile phone use among teenagers (11–21 y.o.) predicted a higher incidence of depressive and anxiety symptoms [12,13] as well as higher hyperactivity/inattention and conduct problems [14,15]. Similarly, some cross-sectional studies have shown that greater mobile phone use among children and adolescents (5–19 years) was particularly related to concentration problems, attention problems, hyperactivity symptoms, and conduct problems [10,11].

We conducted a cross-sectional study to analyze the association between mobile phone screen exposure and cognitive health in adolescents as well as the impact that other quantitative variables (i.e., biological markers such as sex or BMI, other lifestyle factors, and maternal education) have on this association. The goal of this study was to understand how much young adults are influenced by these indicators and to recognize the impacts caused to their cognitive health due to excessive mobile phone screen exposure. Thus, our main hypothesis was that higher screen use in adolescents would be related to poorer cognitive health.

## 2. Materials and Methods

### 2.1. Study Design and Participants

This is a cross-sectional study using baseline data from an existing database provided by the WALNUTs project [16]. The original aim of WALNUTs was the assessment of a nutritional intervention based on daily walnut intake and its impact on cognitive function. A secondary aim was the study of lifestyle factors and their associations with cognition. This is a cohort sample of healthy teenagers (11 to 16 years old) of both sexes recruited during 2016–2017 from several high schools in Barcelona (Spain) and equally distributed in each city district. The students from the 1st to 3rd grades of secondary education from 12 high schools were invited to participate in the project, which was explained to them. The students that volunteered to participate in the study and their guardians signed a consent form. Criteria were established to rule certain participants out. These included students who presented food allergies, were participants of other intervention studies, and those who had neurological diseases. The original sample was 722 participants (24% of the total students invited from the 12 schools), but the eligible participants in the present study were those who reported having access to a personal mobile phone (*n* = 632). The trial was approved by the Clinical Research Ethics Committee of Parc Salut Mar (approval number: 2015/6026/I). Written informed consent to participate in this study was provided by the participants’ legal guardians/next of kin.

### 2.2. Sociodemographic, Clinical, and Lifestyle Data

At baseline, in a face-to-face visit, a fieldwork technician administered questionnaires to the adolescent participants, while other questionnaires were given to be filled out at home by the parents and the adolescent participants and returned to us through the school teachers. During the first visit, data were collected from each participant regarding their eating habits, weekly physical activity frequency, sleeping habits, and sociodemographic information as well as the mother’s educational background (MEB). We also assessed the adherence to the Mediterranean diet by creating a score based on the consumption of fruits, vegetables, legumes, seafood, cereals, nuts, dairy, and olive oil (kidmed test) [16,17]. Data were obtained from a short true/false 16-item questionnaire that was modified to a relative score with a final total score of 12. A score of 3 or less equals low adherence, a score from 4 to 7 equals medium adherence, and a score of 8 or greater equals high adherence. Due to a very small portion of participants with low adherence (*n* = 18), the variable was reclassified into two categories (1–7 = normal adherence and 8–12 = high adherence). Height, weight, and waist circumference were measured by standard methods (height was measured using a SECA 214 stadiometer, weight was measured by SECA 770 weighing scales, and waist circumference was measured with SECA 201 tape), and the weight/height^2^ body mass index (BMI) was calculated.

Data regarding their mobile phone screen exposure (MPSE) were also collected under the question “how many minutes per day do you use your phone?”, and this question was repeated each time to identify what they used their phones for (i.e., games, email, messages, and social media). Time spent on the mobile phone was the sum of all these different phone activities and was divided into three categories based on tertiles: low (L) (less than 9 min per day), medium (M) (from 9 to 20 min per day), and high (H) (more than 20 min per day). Several activities conducted in front of mobile phone screens were identified and combined to make this variable of time spent using a mobile phone. These included: games, email, messages, social media, netsurfing, and others.

### 2.3. Neuropsychological and Behavioral Testing for Primary Endpoints

Several computer-based neuropsychological tests were assessed at the schools, following a strict protocol and with the supervision of an experienced psychologist. The school rooms for testing were previously selected, and the fieldwork technicians prepared the laptops and related equipment. One-hour sessions were conducted in groups of 15 adolescents by three examiners supervised by the psychologist, who explained the test instructions before starting each session. Students were provided with headphones to minimize distractions and contact with each other. Three cognitive outcomes at baseline were selected for this study: attention function, working memory, and fluid intelligence.

The first primary outcome measurement was the Attention Network Test (ANT), a computer-based test designed to assess the attentional function and the integrity of the three attentional networks [18]: alerting, the ability to produce and maintain optimal vigilance and performance during tasks; orienting, which involves shifting attention to sensory stimuli; and executive attention, which involves detecting and resolving shifting attention to sensory stimuli. The test consisted of the appearance of five arrows on the screen, and participants had to indicate the direction of the central arrow as fast as possible. A detailed description of the ANT can be found elsewhere [19]. The ANT also measures accuracy and hit reaction time standard error (HRT-SE), where a lower score indicates good attention performance with consistent reaction times, and the higher the score, the higher the student’s levels of inattention. For this study, we used the HRT-SE (milliseconds) score to indicate inattentiveness.

The second measurement was the N-back test, which was used to assess working memory (WM) [20]. This is also a computerized test that studies the retention of a previously presented stimulus using series of numbers. The 1-back test consists of the participant having to remember the previous stimulus, the 2-back test, on the other hand, requires the participant to respond when the stimulus corresponds to the one presented before the previous stimulus, while the 3-back test requires two previous stimuli. For the purpose of this study, the 3-back test was used due to the ceiling effects found in the 1-back and 2-back tests among healthy teenagers. The more correct answers, the higher the score, and the detection of a more accurate performance is noted; d prime was the outcome selected for this test (d’).

The third measurement, used to assess fluid intelligence (FI), was the Primary Mental Ability test (PMA-R), which analyzes seven abilities: word fluency, verbal comprehension, spatial visualization, number facility, associative memory, reasoning, and perceptual speed [21]. In this case, we only used one of these tests in the assessments. The test used was reasoning, and it consisted of choosing a letter from a set of six possible alternatives, underlying a given sequence of letters. Thus, it addresses inductive reasoning based on letter patterns. The total score is the number of correct item responses. A higher score also indicates higher FI abilities.

### 2.4. Statistical Analysis

Associations between MPSE and cognitive outcomes were assessed using three multivariable linear regression models. A linear regression model is used to assess the association between an independent variable and the outcome, and it is able to statistically control for the potential confounding effects of the selected covariables included in the regression model. MPSE was evaluated as a categorical variable divided into three categories (low, medium, and high). Cognitive outcomes were evaluated as continuous variables. First, a bivariate relation and distribution were studied between our main exposure and the covariates, which were further studied with the cognitive outcomes using an ANOVA. In the regression models, the low-MPSE category was considered the refence group, and the other two MPSE category groups were always compared with the low category group. Next, a crude bivariate linear regression model was carried out between the MPSE categories and the cognitive outcomes. Last, two multivariate linear regression models were built: first, a model adjusted for some confounders, i.e., sex, age, BMI, and the mother’s educational background, and second, a fully adjusted multivariate model with all confounders and potential intermediate factors included (sleep hours, Mediterranean diet scores, and physical activity frequency). Sex (male and female) and BMI (normal and overweight) were included in the analyses as categorical variables, while age was studied as a continuous variable (12 to 16 years old). MEB was divided into two categories: mothers who studied up to high school and mothers that had completed a bachelor’s degree or more. As intermediary variables, lifestyle practices such as physical activity frequency (PAF) (one, two, three, or more than three times per week), sleep hours (less than 8 h vs. 8 h or more per day), and Mediterranean diet scores (normal and high) were analyzed as categorical variables. The independence of the observations, residual distribution, homoscedasticity, and multicollinearity of every regression model were always checked.

All statistical analyses were conducted using STATA 13. Two-tailed *p*-values below 0.05 were considered statistically significant for all analyses.

## 3. Results

The baseline characteristics of the study population by exposure group are shown in Table 1. Participants had an equal gender distribution in the low-exposure group, whereas in the medium- and high-exposure groups females were the predominant gender (59% and 58%, respectively). The average age of children in the study was 13.75 (0.93), 13.91 (0.96), and 14.01 (0.95) years for the low-, medium-, and high-exposure groups, respectively. Most adolescents had a BMI considered to be normal in all groups, although in the high-exposure group the percentage of overweight was somewhat higher (33%). The sleep hours for most participants were 8 or higher in all groups, and they had normal adherence to the Mediterranean diet. Mean differences between exposure groups were found for age, sleep hours, and Mediterranean diet scores (*p*-value < 0.05), indicating possible relations between the exposure and these variables.

Table 2 shows the descriptive results for the primary outcomes according to the MPSE groups. Students had about the same mean scores for the PMA-R and N-back tests in all groups, but the ANT scores differed between them, being 143.84 ms for the low-exposure group, 157.76 ms for the medium-exposure group, and 153.95 ms for the high-exposure group. No significant differences were found between the exposure groups.

When analyzing the association between screen-time exposure and the primary outcomes (Table 3), we found that for ANT HRT-SE scores in crude models there was a 14.9 ms (95% CI = 0.6; 29.3) increase from the low-MPSE (reference) group to the medium-MPSE group and an 11.1 ms (95% CI = 2.8; 25.0) increase in the high-MPSE group. Furthermore, after adjusting for the confounders, the coefficient of the medium-MPSE group increased to 17.6 ms (95% CI = 3.4; 31.7), and it was statistically significant. No significant association was observed in the high-MPSE group; however, the coefficient was of a similar magnitude in the crude model. Regarding fluid intelligence (PMA-R, the number of correct items) and working memory (N-back, d’), there were no significant associations in either exposure group for both the crude and the adjusted analyses. None of these significant associations passed the Bonferroni correction for multiple statistical comparisons with a *p*-value lower than 0.005.

By repeating the models of Table 3 and further adjusting for potential intermediate factor variables (FAA), a slightly increased relationship between inattentiveness and the MPSE groups was observed (Table 4), with both groups (medium and high) being statistically significant. Again, the fluid intelligence and working memory scores showed no significant associations across the exposure groups. Again, none of these significant associations passed the Bonferroni correction with a *p*-value lower than 0.005.

## 4. Discussion

In this cross-sectional study, we mainly observed that there is a positive association between mobile phone screen exposure and inattentiveness among healthy teenagers. This association holds similarly between the crude models and the models that were fully adjusted by the selected confounder factors. Furthermore, adjusting the final models for potential intermediate factors such as sleep duration, Mediterranean diet scores, and physical activity frequency did not change the findings, and we assume that these factors did not explain part of the association of interest. Other important cognitive outcomes, such as working memory and fluid intelligence, did not show any association with mobile phone screen exposure duration.

The main finding to be highlighted from this study is a plausible influence of mobile phone screen exposure on attention. Moreover, this association holds when adjusting for confounding variables. This follows the same patterns that previous studies have mentioned when analyzing the association between attention and screen exposure [22]. In accordance with our results, a cross-sectional study in 6- to 16-year-old students found increased attention problems, among other symptoms (i.e., headaches, fatigue, and concentration problems), in students who had used mobile phones for more than 10 min/day compared to those who never used phones [23]. Nonetheless, this notion should be confirmed in future randomized controlled trials in healthy teenagers. However, these results are the first step towards studying a possible association between screen exposure and attention impairment. In fact, a longitudinal study among 11- to 15-year-old adolescents found a robust association between reported daily digital technology usage and same-day symptoms of attention deficit hyperactivity disorder and conduct disorder [14]. Further, a thorough study focused on young adults (21–32 y.o.) revealed that heavy smartphone use was related to impaired attention, lower numerical processing capability, alterations in social cognition, and decreased excitability of the right prefrontal cortex (rPFC) [24]. Although these findings refer to young adults, the brain is still maturing throughout adolescence. Thus, the effect of mobile phone usage in teenagers may offer an even greater risk. In contrast, according to our results, mobile phone screen exposure does not seem to be related to fluid intelligence or working memory, which concurs with the findings of the aforementioned study [24]. Some other studies have reported an adverse association with general cognitive scores among children [3,9,22]. However, there is the limiting fact that there is a restrictive amount of literature studying the association between MPSE and the cognitive outcomes selected for this study. Further, most studies seem to be focused on young children, whereas adolescents are not being equally studied. The fact that the WHO has recently established screen-time exposure limits for babies and children up to the age of 5 is an example of how most of these efforts have been focused on young children, as it is a critical phase in one’s life where rapid physical and cognitive development takes place, and lifestyle behaviors learned during this period can be beneficial or detrimental throughout the individual’s life course [25]. This highlights the importance of further studying the effect of screens on cognition in adolescents since, at this stage of their development, the brain undergoes substantial structural and functional changes, where complex cognitive functions are fully developed, such as attention [26,27,28], especially considering that adolescence is a stage in which the use of screens tends to increase [8,9].

Additionally, we can infer some other possible relations from our descriptive tables. First, we can see how females are the ones that reported to have higher mobile phone screen exposure than males. However, this should not infer that their overall exposure to screens is actually higher since males have been found to spend more time on videogames [3], so their preference for screen exposure may simply be different. Age also seems to have a relation with mobile phone screen exposure, as older adolescents reported to have higher MPSE. It would be useful to know about home habits, as it is possible that rules controlling MPSE in younger children are more enforced than with older children since adolescence represents a time where the individual gains greater independence and assumes responsibility for daily habits [29]. Although no statistical significance was reported between the exposure groups, it is also important to note the trend for BMI in students. The largest cluster of overweight adolescents was exposed to more screen time, while the largest cluster of normal weight adolescents was in the low-exposure group. This matter correlates with other studies reporting that higher screen exposure is associated with a sedentary lifestyle, which in turn leads to the prevalence of overweight and obesity [30,31,32,33].

Second, an interesting finding is that adolescents that claimed to sleep less than 8 h reported a high MPSE, while students that declared more sleep hours per night reported to have less MPSE, which ties in perfectly with studies that reported correlations between sleep and screen exposure [6,34,35,36]. Thus, the results show that sleep hours have a significant association with higher MPSE, from which we can assume that MPSE may somehow influences circadian rhythms, as other studies have shown [6,34,37], or that sleep problems might lead to increased phone use. Accordingly, it would be beneficial for future studies to take this into consideration to see if there is an impact on the diurnal and nocturnal use of MPSE since bedtime screen exposure is related to lower production of melatonin [37]. On the other hand, there is not a wide variety of studies on the association between the Mediterranean diet and MPSE, although there are more studies related to the association with cravings [38]. However, in the present study we can see how adolescents reporting a higher adherence to the Mediterranean diet do in fact have less MPSE. It would be interesting to further study other lifestyle habits, as it could be assumed that people with high adherence to the Mediterranean diet overall would be more conscious of certain lifestyle choices. Thus, it might not be the diet per se having a direct effect but mainly the family’s conscious lifestyle.

One of the biggest limitations of this study was that our exposure relied on a method involving self-reporting (i.e., questionnaires), which have several limitations that affect both the accuracy and the precision of the measurement (i.e., over- and under-reporting, interviewer bias, incorrect screen exposure estimations, and coding and computational errors). Thus, it was assumed that participants were being truthful with their answers. However, there is a concern that the range of time spent exposed to mobile phone screens reported by students was too low since studies show that 75% of adolescents exceed the amount of recommended leisure screen-time exposure of two hours [8,9], which leads to questioning the truthfulness of the students’ answers regarding the time they spend using their phones. Out of 633 participants that had their own cellphone, only 2 students reported MPSE of 3 h per day. A total of 27 students reported spending between 1 and 2 h on their phones, while the rest reported between 20–58 min per day. This would mean that most students do not exceed the limit of 2 h of daily screen time recommended by the AAP, CPS, and the ADH [3]. It would have been beneficial to have a larger population of students that used their phones for more than one hour per day to obtain a deeper insight into how more time spent exposed to mobile phone screens can impact their cognitive function. Nonetheless, if in fact the answers were truthful and did not underestimate the real exposure, this study shows that even a small amount of MPSE of less than 2 h can impact adolescents’ attention function, raising an alarm that the recommend time might be too lenient, and further studies should be conducted. Indeed, in the case of underreporting mobile phone use, it may have been proportionally similar in each exposure group. Further, a main strength would be that students were equally distributed in several schools, making it possible to assume that the school environment was not a confounding issue to worry about. Another limitation was the small study population. It would have been optimal to have a larger sample size as well as to know other characteristics, such as habits and rules that were practiced at home, to see if these have some sort of effect in the scoring. Further, a limitation to be considered is that the associations were weak and did not pass the multiple test corrections, such as the Bonferroni correction. Therefore, we cannot fully reject that the significant associations may have been found by chance. Moreover, another association weakness is the lack of linearity in the different exposure groups since the teenagers in the middle-exposure and high-exposure groups showed similar coefficients when comparing them with the lowest exposure group. This lack of linearity between the exposure groups suggests an increased chance of false-positive results. Finally, given the cross-sectional nature of this study, we cannot reject reverse causation in the associations between MPSE and cognitive function.

## 5. Conclusions

Overall, our results suggest that screen exposure may affect attention performance in typically developing adolescents. The involvement of several lifestyle factors did not show any influence in the main association. More studies are needed to determine the causality of these associations and to further elucidate the effects of mobile phone screen exposure beyond cognitive function during adolescence. Looking at this under a different light, this study could provide the insight that the recommended guidelines should be revisited, as even an exposure as low as 20 min per day can have detrimental effects on students’ cognitive performance.

## Figures and Tables

**Table 1:** Baseline characteristics of the study population by exposure group and the relation between each covariate and the main exposure (*n* = 632).

Characteristics	Exposure: Daily MPSE					
	*n*	Low (<9 min/day)	*n*	Medium (9–20 min/day)	*n*	High (>20 min/day)
***Biological markers***						
Sex *	224		191		218	
female		108 (48)		112 (59)		126 (58)
male		116 (52)		79 (41)		92 (42)
BMI	223		190		217	
normal		163 (73)		143 (75)		145 (67)
overweight		60 (27)		47 (25)		72 (33)
Age *, mean (SD)	211	13.75 (0.93)	184	13.91 (0.96)	213	14.01 (0.95)
***Maternal***						
MEB	224		191		217	
low		90 (40)		69 (36)		95 (44)
high		134 (60)		122 (64)		122 (56)
***Lifestyle***						
PAF	224		189		217	
Once a week		40 (18)		43 (23)		33 (15)
Twice a week		64 (29)		49 (26)		49 (23)
3 times a week		58 (26)		42 (22)		64 (29)
>3 times a week		62 (28)		55 (29)		71 (33)
Sleep hours *	219		185		216	
less than 8		44 (20)		42 (23)		74 (34)
8 or more		175 (80)		143 (77)		142 (66)
MD scores *	216		186		184	
normal		111 (51)		117 (63)		114 (62)
high		105 (49)		69 (37)		70 (38)

Unless otherwise indicated, data are expressed as the number (percentage) of participants. MPSE, mobile phone screen exposure; BMI, body mass index; MEB, mother’s educational background; PAF, physical activity frequency; MD, Mediterranean diet. * *p*-value < 0.05 obtained through chi square test. Age, being a continuous variable, was analyzed using an ANOVA test to study the correlation between means.

**Table 2:** Cognitive scores by mobile phone screen exposure groups.

Cognitive Outcomes (Mean, (SD))	Exposure: Daily MPSE					
	*n*	Low (<9 min/d)	*n*	Medium (9–20 min/d)	*n*	High (>20 min/d)
HRT-SE (ms) (Inattentiveness)	213	143.84 (66.00)	185	157.76 (75.51)	209	153.95 (76.97)
PMA-R score (number of correct items) (Fluid Intelligence)	213	16.69 (5.71)	183	17.05 (5.62)	209	17.15 (5.36)
N-Back score (d’) (Working Memory)	211	2.08 (0.76)	183	2.13 (0.8)	207	2.04 (0.77)

MPSE, mobile phone screen exposure; HRT-SE, hit reaction time standard error; PMA-R, Primary Mental Ability Test. *p*-values were calculated using ANOVA tests. Significance was set to *p* < 0.05. None of the results were statistically significant.

**Table 3:** Association between mobile phone screen exposure and cognitive outcomes, adjusting and not for confounders.

MPSE	HRT-SE (ms) (Inattentiveness)		PMA-R Score (Fluid Intelligence)		N-Back Score (Working Memory)	
	CA	AA	CA	AA	CA	AA
*N*	607	580	605	578	601	574
Low (<9 min/d)	ref.	ref.	ref.	ref.	ref.	ref.
Medium (9–20 min/d)	14.9 * (0.6, 29.3)	17.6 * (3.4, 31.7)	0.36 (−0.73, 1.47)	−0.15 (−1.25, 0.95)	0.456 (−0.11, 0.20)	0.019 (−0.14, 0.18)
High (>20 min/d)	11.1 * (2.8, 25.0)	12.9 (−0.8, 31.7)	0.46 (−0.6, 1.52)	0.11 (−0.95, 1.18)	−0.428 (−0.19,0.11)	−0.441 (−0.20, 0.11)

MPSE, mobile phone screen exposure; HRT-SE, hit reaction time standard error; PMA-R, Primary Mental Ability Test; *N*, number of subjects with available data. Data are expressed as β coefficients (95% CI) obtained through bivariable linear regression (CA, crude analysis) and multivariable linear regression (AA, adjusted analysis). Cofounders for the AA include sex, BMI, age, and mother’s educational background. * *p*-value < 0.05.

**Table 4:** Association between mobile phone screen exposure and cognitive outcomes, adjusting for intermediate factors.

MPSE	HRT-SE (ms) (Inattentiveness)		PMA-R Score (Fluid Intelligence)		N-Back Score (Working Memory)	
	AA	FAA	AA	FAA	AA	FAA
*N*	580	559	578	557	574	553
Low (<9 min/d)	ref.	ref.	ref.	ref.	ref.	ref.
Medium (9–20 min/d)	17.6 * (3.4, 31.7)	15.8 * (1.4, 30.3)	−0.15 (−1.25, 0.95)	0.09 (−1.12, 1.14)	0.019 (−0.14, 0.18)	0.003 (−0.16, 0.17)
High (>20 min/d)	12.9 (−0.8, 31.7)	14.97 * (0.9, 29.1)	0.11 (−0.95, 1.18)	0.21 (−1.08, 1.12)	−0.441 (−0.20, 0.11)	−0.052 (−0.21, 0.11)

MPSE, mobile phone screen exposure; HRT-SE, hit reaction time standard error; PMA-R, Primary Mental Ability Test; *N*, number of subjects with available data. Data are expressed as β coefficients (95% CI) obtained through multivariable linear regression. AA (adjusted analysis) is adjusted for sex, BMI, age, and mother’s educational background. FFA (fully adjusted model) is further adjusted for sleep hours, Mediterranean diet scores, and physical activity frequency. * *p*-value < 0.05.

## Data Availability

Not applicable.

## References

[1] Lepp A., Barkley J.E., Karpinski A.C. (2014). The relationship between cell phone use, academic performance, anxiety, and Satisfaction with Life in college students. Comput. Hum. Behav..

[2] Lee D., Jackson M. (2017). The Simultaneous Effects of Socioeconomic Disadvantage and Child Health on Children’s Cognitive Development. Demography.

[3] Lissak G. (2018). Adverse physiological and psychological effects of screen time on children and adolescents: Literature review and case study. Environ. Res..

[4] Toh S.H., Howie E.K., Coenen P., Straker L.M. (2019). “From the moment I wake up I will use it⋯every day, very hour”: A qualitative study on the patterns of adolescents’ mobile touch screen device use from adolescent and parent perspectives. BMC Pediatr..

[5] Twenge J.M., Campbell W.K. (2018). Associations between screen time and lower psychological well-being among children and adolescents: Evidence from a population-based study. Prev. Med. Rep..

[6] Twenge J.M., Hisler G.C., Krizan Z. (2018). Associations between screen time and sleep duration are primarily driven by portable electronic devices: Evidence from a population-based study of U.S. children ages 0–17. Sleep Med..

[7] Marques A., Calmeiro L., Loureiro N., Frasquilho D., de Matosabg M.G. (2015). Health complaints among adolescents: Associations with more screen-based behaviours and less physical activity. J. Adolesc..

[8] Babic M.J., Smith J.J., Morgan P.J., Eather N., Plotnikoff R.C., Lubans D.R. (2017). Longitudinal associations between changes in screen-time and mental health outcomes in adolescents. Ment. Health Phys. Act..

[9] Zink J., Belcher B.R., Kechter A., Stone M.D., Leventhal A.M. (2019). Reciprocal associations between screen time and emotional disorder symptoms during adolescence. Prev. Med. Rep..

[10] Girela-Serrano B.M., Spiers A.D.V., Ruotong L., Gangadia S., Toledano M.B., Di Simplicio M. (2022). Impact of mobile phones and wireless devices use on children and adolescents’ mental health: A systematic review. Eur. Child Adolesc. Psychiatry.

[11] Wacks Y., Weinstein A.M. (2021). Excessive Smartphone Use Is Associated With Health Problems in Adolescents and Young Adults. Front. Psychiatry.

[12] Bickham D.S., Hswen Y., Rich M. (2015). Media use and depression: Exposure, household rules, and symptoms among young adolescents in the United States. Int. J. Public Health.

[13] Liu S., Wing Y.K., Hao Y., Li W., Zhang J., Zhang B. (2019). The associations of long-time mobile phone use with sleep disturbances and mental distress in technical college students: A prospective cohort study. Sleep.

[14] George M.J., Russell M.A., Piontak J.R., Odgers C.L. (2018). Concurrent and Subsequent Associations between Daily Digital Technology Use and High-Risk Adolescents’ Mental Health Symptoms. Child Dev..

[15] Schoeni A., Roser K., Röösli M. (2017). Symptoms and the use of wireless communication devices: A prospective cohort study in Swiss adolescents. Environ. Res..

[16] Julvez J., Gignac F., Fernández-Barrés S., Romaguera D., Sala-Vila A., Ranzani O.T., Persavento C., Delgado A., Carol A., Torrent J. (2021). Walnuts, Long-Chain Polyunsaturated Fatty Acids, and Adolescent Brain Development: Protocol for the Walnuts Smart Snack Dietary Intervention Trial. Front. Pediatr..

[17] Rei M., Severo M., Rodrigues S. (2021). Reproducibility and validity of the Mediterranean Diet Quality Index (KIDMED Index) in a sample of Portuguese adolescents. Br. J. Nutr..

[18] Forns J., Esnaola M., López-Vicente M., Suades-González E., Alvarez-Pedrerol M., Julvez J., Grellier J., Sebastián-Gallés N., Sunyer J. (2014). The n-back test and the attentional network task as measures of child neuropsychological development in epidemiological studies. Neuropsychology.

[19] Fan J., McCandliss B.D., Sommer T., Raz A., Posner M.I. (2002). Testing the efficiency and independence of attentional networks. J. Cogn. Neurosci..

[20] López-Vicente M., Forns J., Suades-González E., Esnaola M., García-Esteban R., Álvarez-Pedrerol M., Júlvez J., Burgaleta M., Sebastián-Gallés N., Sunyer J. (2016). Developmental trajectories in primary schoolchildren using n-back task. Front. Psychol..

[21] THURSTONE T.G. (1957). The Tests of Primary Mental Abilities. Pers. Guid. J..

[22] Fu J., Xu P., Zhao L., Yu G. (2018). Impaired orienting in youth with Internet Addiction: Evidence from the Attention Network Task (ANT). Psychiatry Res..

[23] Mortazavi S.M.J., Atefi M., Kholghi F. (2011). The Pattern of Mobile Phone Use and Prevalence of Self-Reported Symptoms in Elementary and Junior High School Students in Shiraz, Iran. Iran. J. Med. Sci..

[24] Hadar A., Hadas I., Lazarovits A., Alyagon U., Eliraz D., Zangen A. (2017). Answering the missed call: Initial exploration of cognitive and electrophysiological changes associated with smartphone use and abuse. PLoS ONE.

[25] (2019). Guidelines on Physical Activity, Sedentary Behaviour and Sleep for Children under 5 Years of Age.

[26] Toga A.W., Thompson P.M., Sowell E.R. (2006). Mapping brain maturation. Trends Neurosci..

[27] Rossi A.F., Pessoa L., Desimone R., Ungerleider L.G. (2009). The prefrontal cortex and the executive control of attention. Exp. Brain Res..

[28] Petersen S.E., Posner M.I. (2012). The Attention System of the Human Brain: 20 Years After. Annu. Rev. Neurosci..

[29] Soleimanpour S., Geierstanger S., Brindis C.D. (2017). Systems of Care and Clinical Practice: Adverse Childhood Experiences and Resilience: Addressing the Unique Needs of Adolescents. Acad. Pediatr..

[30] Keane E., Kelly C., Molcho M., Gabhainn S.N. (2017). Physical activity, screen time and the risk of subjective health complaints in school-aged children. Prev. Med..

[31] Kelly S., Stephens J., Hoying J., McGovern C., Melnyk B.M., Militello L. (2017). Special Section: Council for the Advancement of Nursing Science 2016: A systematic review of mediators of physical activity, nutrition, and screen time in adolescents: Implications for future research and clinical practice. Nurs. Outlook.

[32] Tsiros M.D., Samaras M.G., Coates A.M., Olds T. (2017). Use-of-time and health-related quality of life in 10- to 13-year-old children: Not all screen time or physical activity minutes are the same. Qual. Life Res..

[33] Haskell W., Bull F.C., Andersen L.B., Hallal P.C., Guthold R., Ekelund U. (2012). Global physical activity levels: Surveillance progress, pitfalls, and prospects. Lancet.

[34] Cain N., Gradisar M. (2010). Electronic media use and sleep in school-aged children and adolescents: A review. Sleep Med..

[35] Arora T., Brogli E., Thomas G.N., Taheri S. (2014). Associations between specific technologies and adolescent sleep quantity, sleep quality, and parasomnias. Sleep Med..

[36] Hale L., Guan S. (2016). Screen Time and Sleep among School-Aged Children and Adolescents: A Systematic Literature Review. Sleep Med. Rev..

[37] Cabré-Riera A., Torrent M., Donaire-Gonzalez D., Vrijheid M., Cardis E., Guxens M. (2019). Telecommunication devices use, screen time and sleep in adolescents. Environ. Res..

[38] Shang L., Wang J., Loughlin J., Tremblay A., Mathieu M., Henderson M., Gray-Donald K. (2015). Screen time is associated with dietary intake in overweight Canadian children. Prev. Med. Rep..

